# Resting state electroretinography: An innovative approach to intrinsic retinal function monitoring

**DOI:** 10.3389/fphys.2022.931147

**Published:** 2022-08-26

**Authors:** Mercedes Gauthier, Antoine Brassard Simard, Anna Polosa, Allison L. Dorfman, Cynthia X. Qian, Jean-Marc Lina, Pierre Lachapelle

**Affiliations:** > ^1^ Département de Génie Électrique, École de Technologie Supérieure, Montreal, QC, Canada; ^2^ Department of Ophthalmology and Visual Sciences, Research Institute of the McGill University Health Centre/Montreal Children’s Hospital, Montreal, QC, Canada; ^3^ Department of Ophthalmology, Maisonneuve-Rosemont Hospital Research Centre, University de Montréal, Montréal, QC, Canada; ^4^ Department of Ophthalmology, Centre Hospitalier Universitaire Sainte-Justine Research Center, Montréal, QC, Canada; ^5^ Centre de Recherches en Mathématiques, Montréal, QC, Canada

**Keywords:** electroretinogram, spectral analysis, multifractal analysis, wavelet leaders, retina, resting-state

## Abstract

The electroretinogram (ERG) represents the biopotential evoked by the retina in response to a light stimulus. The flash evoked ERG (fERG) is the ERG modality most frequently used clinically to diagnose and monitor retinal disorders. We hereby present a new method to record spontaneous retinal activity, without the use of a flash stimulus, that we named the resting-state ERG (rsERG). The recordings were done in normal subjects under light- and dark-adaptation and with different background light conditions (i.e., variations of wavelength and intensity). Additionally, rsERG recordings were obtained in five patients with retinopathies. The signals were subsequently analyzed in the frequency domain, extracting both periodic (i.e., frequency peaks) and aperiodic (i.e., background trend) components of the signal. The later was further assessed through a multifractal analysis using Wavelet Leaders. Results show that, irrespective of the recording conditions used, the rsERG always includes the same 90 Hz component; a frequency component also present in the fERG response, suggesting a retinally-intrinsic origin. However, in addition, the fERGs also includes a low-frequency component which is absent in the rsERGs, a finding supporting a retinally-induced origin. Comparing rsERGs with fERGs in selected patients with various retinal disorders indicates that the two retinal signals are not always similarly affected (either as a result of underlying retinal pathology or otherwise), suggesting an added value in the assessment of retinal function. Thus, the rsERG could have a similar role in clinical visual electrophysiology as that of the resting-state EEG in neurology namely, to quantify changes in spontaneous activity that result from a given disease processes.

## 1 Introduction

The electroretinogram (ERG) represents the biopotential generated by the retina in response to a light stimulus (McCulloch et al., 2015). To date, the flash-evoked ERG (or fERG) represents the only tool available to assess the functional integrity of the retina, normal or diseased (Heckenlively and Arden, 2006; Lam, 2005). The fERG thus falls in the category of Evoked Potentials (EP) since a stimulus (i.e., a flash of light) is required to evidence it, unlike the resting-state electroencephalogram (rsEEG) or the electrocardiogram (ECG) which represent the recording of ongoing (or intrinsic) spontaneous electrical activity. EPs are therefore a man-made construct that allow us to artificially assess the functional integrity of neural structures and/or pathways. To be diagnostically relevant, the method used to generate, record and analyze a given EP from a patient must be clearly defined and compared to normative data gathered using an identical approach. These methodological procedures must therefore be standardized (see fERG standard in McCulloch et al. (2015), for example) if universality is sought; a task far more complex compared to the standardization of ongoing electrical potential recordings like the EEG or ECG. The purpose of this study is therefore to investigate if it is possible to disenfranchise the recording of the ERG from the flash of light that triggers it in order to obtain diagnostically meaningful retinal electrical activity without having to rely on a light synchronizer.

Herein, we present an innovative strategy aimed at assessing the non-evoked, spontaneous (i.e., intrinsic or autogenous) electrical activity of the retina, that we named the resting-state ERG (rsERG). This new approach parallels the resting-state EEG, which studies the brain activity at rest (Snyder and Raichle, 2012; Babiloni et al., 2020; Li et al., 2020), and contrasts with the evoked response potential (ERP) of the EEG, which are recorded in response to the execution of a given task or use of a triggering stimulus. Thus, the ERP is to the brain what the fERG is to the retina. These brain-retina functional analogies (i.e., rsEEG vs. rsERG and ERP vs. fERG) are of particular significance considering that the brain and retina were shown to be nearly identical from a cytoarchitecture and functional point of view [and even presented as an approachable part of the brain by Dowling (1987)]. To our knowledge, this study is first to report the recording and analysis of the intrinsic human retinal activity.

Based on the above retina-brain similarities, the rsERG signal will be analyzed using methodologies previously applied to the EEG, such as that detailed in Donoghue et al.’s (2020) study which showed the importance of disentangling the periodic (which emphasizes frequency peaks in the spectrum) and the aperiodic (or scale-free activity) components of neuronal signals. In the present study, we also analyzed these two components, where the periodic component was assessed by means of a spectral analysis of the data and the aperiodic component was modeled with a power law relationship that organizes the various temporal scales as $1/f^{\alpha }$ , an approach also used in EEG (Donoghue et al., 2020; Lina et al., 2019; Gadhoumi et al., 2015). Of note, the aperiodic component was previously shown to yield valuable information as it reveals the spectral organization of the EEG signal, without having to rely on a specific scale or frequency (Ma et al., 2005; He et al., 2010; Zorick and Mandelkern, 2013). For example, changes in behavioral or cognitive states have been shown to modify the underlying fractal pattern of the EEG signal (Gadhoumi et al., 2015; He et al., 2010; Zorick and Mandelkern, 2013). However, there is not typically a unique value for the scaling exponent α characterizing a given biopotential signal, as they generally exhibit a wide range of such exponents. Consequently, using the multifractal formalism allows one to better describe the aperiodic component, as it models the scaling property as a collection of such exponents rather than a single α value. The Wavelet Leaders multifractal formalism (Jaffard, 2004; Jaffard et al., 2006; Wendt and Abry, 2007; Wendt et al., 2007) provides a robust estimator of Hölder exponents spectrum. This framework describes the sets of the exponents present in the signal and characterizes, locally, the $1/f^{\alpha }$ spectrum. Here, we will use two values to describe the multifractal spectrum: H_m_ (the main Hölder exponent) and D (the dispersion of the exponent). Describing the aperiodic component as such has been shown to be a robust mean to estimate the complexity of various biopotentials (Lina et al., 2019; Gadhoumi et al., 2015). Finally, we have previously shown that a scale-free behavior exists in the human photopic fERG (Gauvin et al., 2016; Gauvin et al., 2015), and our assumption here is that this characteristic also holds true even when the retinal response is not triggered/evoked by a flash of light.

## 2 Methods

### 2.1 Subjects and recording material and procedure

The experimental protocol described below was approved by the Institutional Review Board of the Montreal Children’s Hospital and in accordance with the Declaration of Helsinki. fERG and rsERGs were recorded using a Ganzfeld (full-field) stimulator (ColorDome full-field stimulator; Diagnosys Espion system; Diagnosys LLC, Lowell, MA) in 2 different groups of normal subjects: seven subjects in Group 1 (4 females, three males; average age: 24.0 ± 4.5 years) and 15 subjects in Group 2 (12 females, three males; average age: 38.3 ± 15.1 years). These were separated in two groups to avoid the fatigue of our subjects. Given that we are essentially recording baseline noise variations, we felt that exposing subjects to lengthy recording sessions could potentially compromise the quality of our results. Thus, results between the two groups were not compared as they underwent different recording protocols (see Section 2.1.2). Additionally, five patients affected with different retinopathies were also included in this study (3 females, 2 males; average age: 54.0 ± 14.2 years). We elected not to disclose the name of the retinopathy (or any clinical information) to prevent unsupported associations between a given disease process and the resulting rsERG results, associations that might be possible only with a larger population of patients. Rather, our goal was simply to show that a retinal anomaly can impact the rsERG like (or not) the fERG and/or the mfERG. All subjects (normal and patients) went through a complete ophthalmological examination (including: visual acuity measure, optical coherence tomography, visual field, intraocular pression, etc.) by one of the co-authors (CXQ) who is a certified ophthalmologist, in order to confirm the status of the retina (normal or diseased).

The retinal responses were recorded following our previously published method (Woo et al., 2017; Vatcher et al., 2019) and the ISCEV standards (McCulloch et al., 2015). A representation of the electrode setup used can be found on Supplementary Figure S1. DTL fiber active electrodes (27/7 XStatic® silver-coated nylon conductive yarn, Sauquoit Industries, Scranton, PA, United States) were placed deep in the inferior conjunctival bags of each eye, while the ground and reference skin electrodes (Grass gold cup electrodes filled with Ten 20 electrode conductive cream; Natus Neurology) were respectively placed on the forehead and external canthi. After pupil dilation (tropicamide 1%) the subjects were positioned facing the Ganzfeld with their chin on the chin rest and instructed to stare at a fixating red LED light. Photopic fERGs and rsERGs were first obtained after a 10-min period of adaptation to background light (30 cd m^−2^, white light). This was then followed by a 20-min period of dark adaptation to record the scotopic fERGs and rsERGs. Multifocal ERGs (mfERGs) were also recorded after a 10-min period of light adaptation. Both eyes of normal subjects were recorded and results obtained from the left and right eyes were averaged to yield a single data point. However, only one eye is reported per patient. Following the recordings, the signals were exported and subsequently analyzed in Matlab R2021a (MathWorks, Natick, MA, United States).

#### 2.1.1 Flash evoked ERG recording Protocol

**FIGURE 1:**
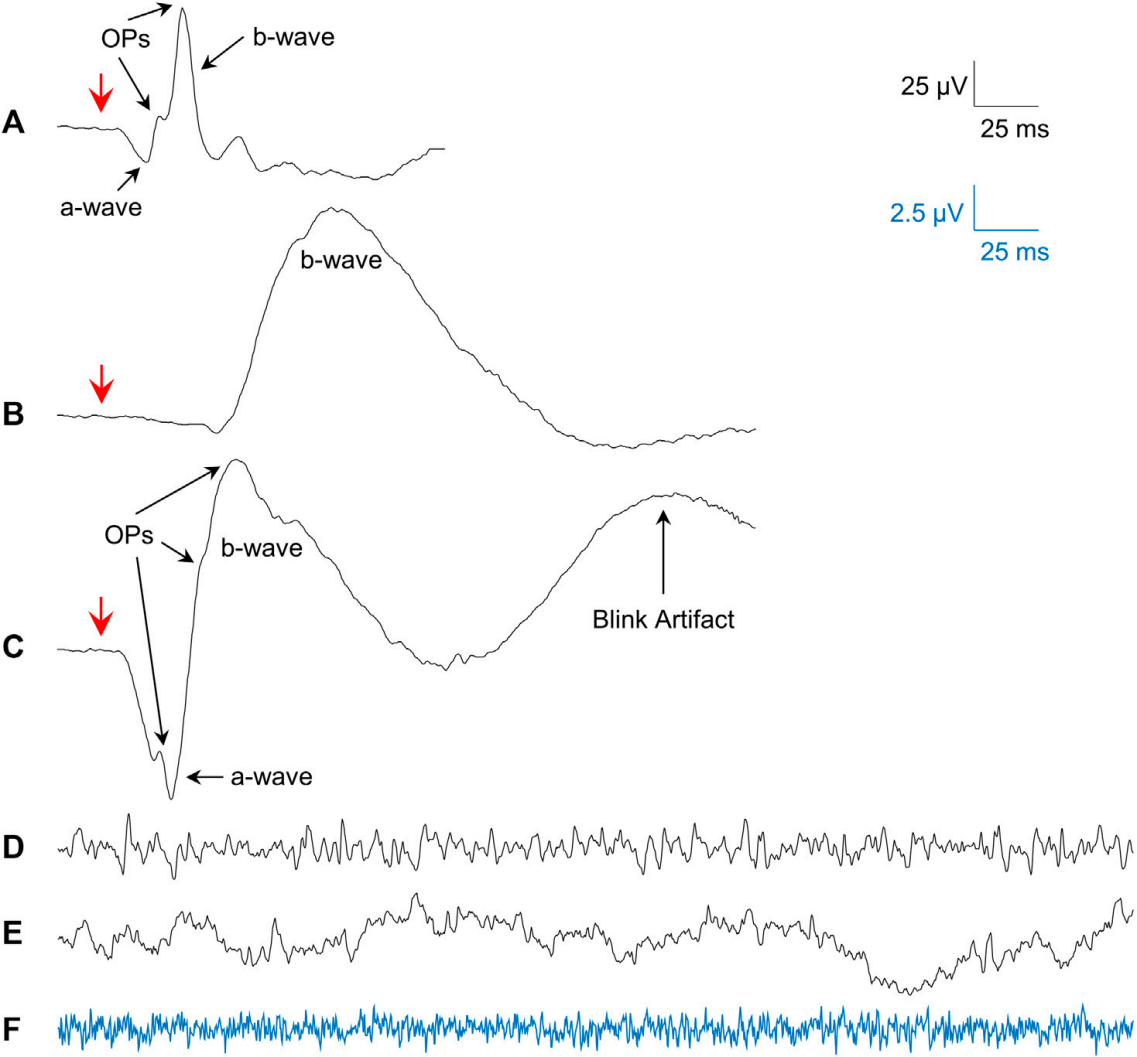
Examples of the signals studied. **(A)** Mean photopic 3.0 cd s m^−2^ fERG from all subjects. **(B)** Mean scotopic 0.005 cd s m^−2^ fERG (pure-rod) from all subjects. **(C)** Mean scotopic 1.0 cd s m^−2^ fERG (mixed rod-cone) from all subjects. **(D)** Single sample of a photopic rsERG with 30 cd m^−2^ white background. **(E)** Single sample of an EEG (forehead electrode) with 30 cd m^−2^ white background. **(F)** Single sample from electrode in saline with 30 cd m^−2^ white background (amplified 4 times; blue signal matches blue calibration bar). Red arrows in **(A–C)** show flash onset.

Averages of photopic (flash intensity: 3 cd s m^−2^; rod desensitizing background light: 30 cd m^−2^ white light; interstimulus interval: 1,500 ms; pre-stimulus baseline: 20 ms) and scotopic (20 min of dark-adaptation; flashes of 0.005 and 1 cd s m^−2^; interstimulus interval: 10 s; pre-stimulus baseline: 20 ms) fERGs (sampling frequency: 3413.33 Hz; bandwidth: 1.25–1,000 Hz) were obtained from all our subjects and patients. Representative examples of the fERG waveforms obtained are shown in Figure 1A (photopic fERG), Figure 1B (scotopic pure rod fERG) and Figure 1C (scotopic mix rod-cone fERG).

#### 2.1.2 Resting-state ERG recording Protocol

Although the recording of the intrinsic retinal electrical activity has never been reported before, we felt that using an approach similar to that used for the fERG (i.e., with the active electrode touching the eye) would be acceptable, given that both techniques are aimed at assessing retinal function. By definition, the rsERG represents the intrinsic retinal activity, free from any sort of external stimulation. Although it is not an activity evoked by a flash of light, we postulated that it could be modulated by varying the background light and/or state of retinal adaptation. Consequently, rsERGs (sampling frequency: 3606 Hz; bandwidth: 0–300 Hz; signal length: 1,020 ms; 3 to 10 individual responses) were obtained as follows. Group 1 subjects were exposed to red (630 ± 20 nm), green (520 ± 35 nm), blue (470 ± 20 nm), white background lights (30 cd m^−2^; LED lights) and finally, dark (light OFF) conditions, respectively. In order to ascertain that the rsERG signal originated from the retina (and not from the brain or of an extraneous electrical source such as the electrode/recording system noise), the above protocol was also used with: 1- the three recording electrodes (i.e., DTL, reference and ground electrodes) immerged in a beaker of saline placed in front of the Ganzfeld (at the same level as normal eye position to assess possible noise contamination from the electrode/recording system), and 2- the active electrode pasted on the forehead of one of the subjects (to record an EEG signal) with the ground and reference placed on the earlobe and external canthi, respectively. Tracings that included evidence of contamination (i.e., blinks, eye or head movements, etc.) were discarded.

Group 2 subjects were exposed to varying intensities of background light in both light and dark-adapted conditions. The background light intensities used were of 0, 1, 20 and 30 cd m^−2^ (white light) in light-adapted conditions, and of 0, 0.0005, 0.001, 0.005, 0.05, and 1 cd m^−2^ (Rod-color light from Diagnosys LLC, Lowell, MA, made from the green and blue LEDs) in dark-adapted conditions. Of note, the fERGs were recorded in this group, before recording the rsERGs in each condition.

#### 2.1.3 Multifocal ERG recording protocol

To assess the macular function of the pathological subjects, we also recorded the mfERGs in accordance with the ISCEV standards (Hoffmann et al., 2021). The test uses a 50° stimulus array composed of 61 hexagons centered at the fovea, enabling the segmentation into 61 contiguous ERG responses produced within that region (Hoffmann et al., 2021). The subjects were placed in front of the stimulus array and asked to stare at a fixating point at the center of the screen. The mfERGs were then recorded (maximal luminance: 500 cd s/m^2^, minimal luminance: 0 cd s/m^2^; background: 250 cd s/m^2^; Bandwidth: 10–100 Hz; eight steps lasting 30 s each) in each subject/patient after a 10 min period of light-adaptation, as per a previously published method of ours (Vatcher et al., 2019; Beneish et al., 2021).

### 2.2 Spectral analysis

Spectral analysis (SA) of the fERG and rsERG was done using two different methods, as the first is more oscillatory while the second more stochastic. For the averaged fERGs responses, we used the fast Fourier transform (FFT) with a spectral resolution of 1.7 Hz as previously published by us (Gauvin et al., 2014; Gauthier et al., 2019). Of note, when a blink artifact was present in the signal, it was removed and replaced by baseline noise (i.e., padding). For the rsERGs, we used Welch’s overlapping segment averaging method, which reduces the variance in the power spectrum and thus is more robust for noisy signals such as this, though the resulting spectral resolution is larger (Hayes, 1996). The power spectral density (PSD) was thus estimated on each sweep with Welch’s method (Hamming window; window size: 512 points; no-overlap size: 50 points; spectral resolution: 7 Hz).

From the power spectrums obtained, we can identify periodic and aperiodic components (Donoghue et al., 2020). The periodic components are the peaks which can be seen in the frequency spectrums. The aperiodic component is the background activity in the spectrum. As the power spectrum p(f) is dominated by this aperiodic component, it can be modeled as:

$$p\left(f\right)=C/f^{\alpha }$$ (1)

**FIGURE 2:**
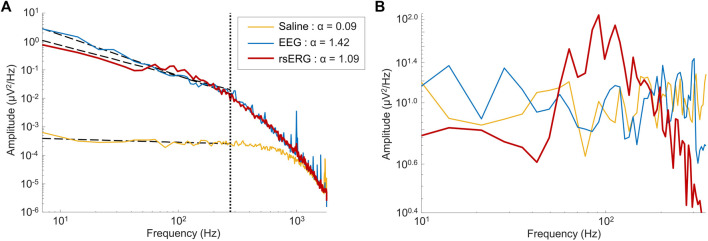
Comparing signals of different sources. **(A)** Mean frequency spectrums depicted on a log-log scale for signals of 1 subject coming from three locations: on the eye (rsERG; red tracing), on the forehead (EEG; blue tracing) and in saline (neighboring electrical noise; yellow tracing). Of note, the vertical dotted line shows the upper limit of data points used in the linear regression, i.e., data points from 7 Hz to 275 Hz, while the other three dotted lines represent each linear regression. The legend displays the α exponent estimated from the linear regression (i.e., the negative slope value), used to remove the background $1/f^{\alpha }$ activity from the frequency spectrums in **(A)** to get the frequency spectrums in **(B)** (zoomed).

This is true when the log-log representation of the spectrum is mostly linear, which happens here between 7 and 275 Hz (0 Hz being the DC shift; Figure 2A). To estimate the values of constant C and the exponent α in this region of the power spectrum, a linear regression ( αf+C ) is made on the log of the power spectrum [ log(p(f)) ] and the log of the frequencies [ log(f) ]. Figure 2A shows examples of these linear regressions (dashed lines), made on the frequency spectrum of each signal, up to the horizontal cutoff at 275 Hz (dotted line) With this linear regression, the $1/f^{\alpha }$ background activity (or aperiodic activity) was removed from the power spectrum to better visualize the underlying periodic activity $p_{o}\left(f\right)$ (see example of resulting spectrum in Figure 2B). This was done by subtracting the estimated linear regression from the power spectrums in the log-log space, which amounts to filtering out the aperiodic component to be left with the periodic component $p_{o}\left(f\right)$ , such that:

$$p_{o}\left(f\right)=f^{\alpha }p\left(f\right)$$ (2)

### 2.3 Scale invariance

As shown above, fitting a linear regression in the log-log scale aims at finding a $1/f^{\alpha }$ behavior in the signal. This enables us to estimate the scale invariance (SI) in the signal, which is the characteristic of time signals with similar statistical properties at different time scales (or frequency). In this case, analyzing the ERG’s SI amounts to calculating the power law relationship that exists between scales (or frequencies) (Gadhoumi et al., 2015; Varsavsky et al., 2011). However, this simple slope estimate is not the best approach to do so, as the slope may vary throughout the frequency domain (i.e., the power spectrum in log-log scale does not show a perfect linear relationship). Because of this, the linear regression was not a perfect match, even though we controlled for its variation by selecting a small segment of the spectrum. A better way would then be to have multiple values expressing this scaling behavior of the data seen in the frequency domain.

To do so, one could also use the Hölder exponent *h*, which characterizes the signal’s local regularity at each time point, where an *h* close to 0 has irregular trajectories (i.e., anti-persistent process) and a larger *h* (close to 1) has smoother ones (i.e., persistent process or long-range dependency). An *h* of 0.5 would be the cutoff between both different signal processes. If the collection of *h* exponents has the same value for the whole signal, this signal is said to be monofractal. If on the other hand the signal has a distribution of varying *h* values, then this signal would be multifractal. Thus, a better way of estimating the signal’s SI is with a multifractal analysis, which does not estimate the SI as a simple constant (as with the linear regression method above) but a whole function describing the collection of all Hölder exponents *h*. For more information on how the $1/f^{\alpha }$ scale-free behavior of the signal is related to the Hölder exponent, see Supplementary Material Annex SI.

### 2.4 Multifractal analysis using the wavelet leaders approach

The Multifractal analysis was done using the freely-accessible *Wavelet Leader and Bootstrap based MultiFractal analysis* (WLBMF) Matlab toolbox, implementing the Wavelet Leaders multifractal formalism developed by Wendt et al. (2007).

This approach estimates the set of local Hölder exponents by estimating a local power law from a modified discrete wavelet transform (DWT) termed the Wavelet Leaders (Jaffard, 2004; Jaffard et al., 2006; Wendt and Abry, 2007; Wendt et al., 2007). Doing so gives a statistically robust and reliable way of estimating the multifractal spectrum.

Using the Wavelet Leaders approach, we estimated the set of Hölder exponents in the signal. The distribution of these exponents was characterized with two values used in this study: the main (or most prevalent) Hölder exponent termed $H_{m}$ , and the dispersion D of these exponents around $H_{m}$ . For more information on how this is calculated and used here, see Supplementary Material Annex SII.

### 2.5 Statistical analysis

Mean values and standard deviations were calculated for each testing parameter. Statistical significance (*p* < 0.05) was determined with t-tests, either one-sampled (to determine statistically negative D values), paired-sampled (between different conditions in the same group) t-tests using Matlab R2021a (MathWorks, Natick, MA, United States). Finally, we compared the morphologies of the rsERG frequency spectrums of normal and pathological subjects, by calculating the correlation coefficient (r^2^; Matlab, *p* < 0.05). This was done only for the frequencies around the frequency peak (i.e., between 14 and 148 Hz).

## 3 Results

### 3.1 Supporting evidence of a retinal origin for the resting-state ERG

Given that a technique to record the intrinsic retinal electrical activity has never been presented before, the first step was to make sure the rsERG obtained using our approach originated from the retina and not from other electrical sources, such as the brain or even electrical noise (from the electrode and recording system). Figure 1 presents examples of these signals, i.e., rsERG signals (Figure 1D), forehead EEG (Figure 1E) and neighboring electrical noise (Figure 1F). The original frequency spectrums of these different signals (tested sequentially, in one subject only) are shown in Figure 2A, where the dashed lines represent the linear regressions made to calculate the α exponents of the $1/f^{\alpha }$ background activity. However, this slope is only calculated within the linear portion of the graph (i.e., between 7 and 275 Hz, 0 Hz being the DC shift; see dashed line limit in Figure 2A) and is therefore not representative of the whole signal (i.e., peaks in the spectrum can affect the linear regression).

In contrast, the neighboring electrical noise tracing (yellow) behaves more like a low-amplitude white noise given that: 1- it is the smallest signal recorded [approximately 2 log units smaller than the other two as per Figure 2A, or 1 log unit smaller in amplitude as per tracings Figure 1 (calibration bar 10 times smaller for tracing F)]; 2- it does not have a $1/f^{\alpha }$ background behavior, its slope being almost 0 (α = 0.09). On the other hand, the other two signals are biopotentials and thus have a $1/f^{\alpha }$ background behavior, which was removed from the frequency spectrums in Figure 2B, to better appreciate the frequency peaks present. It is in this latter periodic component that a clear difference can be observed between the EEG and rsERG signals, where the rsERG (red tracing) shows a peak in amplitude at ∼90 Hz, following a rise which started at ∼40 Hz, while the EEG frequency spectrum (blue tracing) never discloses a significant peak throughout (as expected given the resting-nature of this EEG). Furthermore, the estimated slope roughly describing the aperiodic component shows a difference between the rsERG (α = 1.09) and EEG (α = 1.42).

### 3.2 Attempt at modifying the frequency spectrum signature of the resting-state ERG signal with the modulation of the luminous environment

**FIGURE 3:**
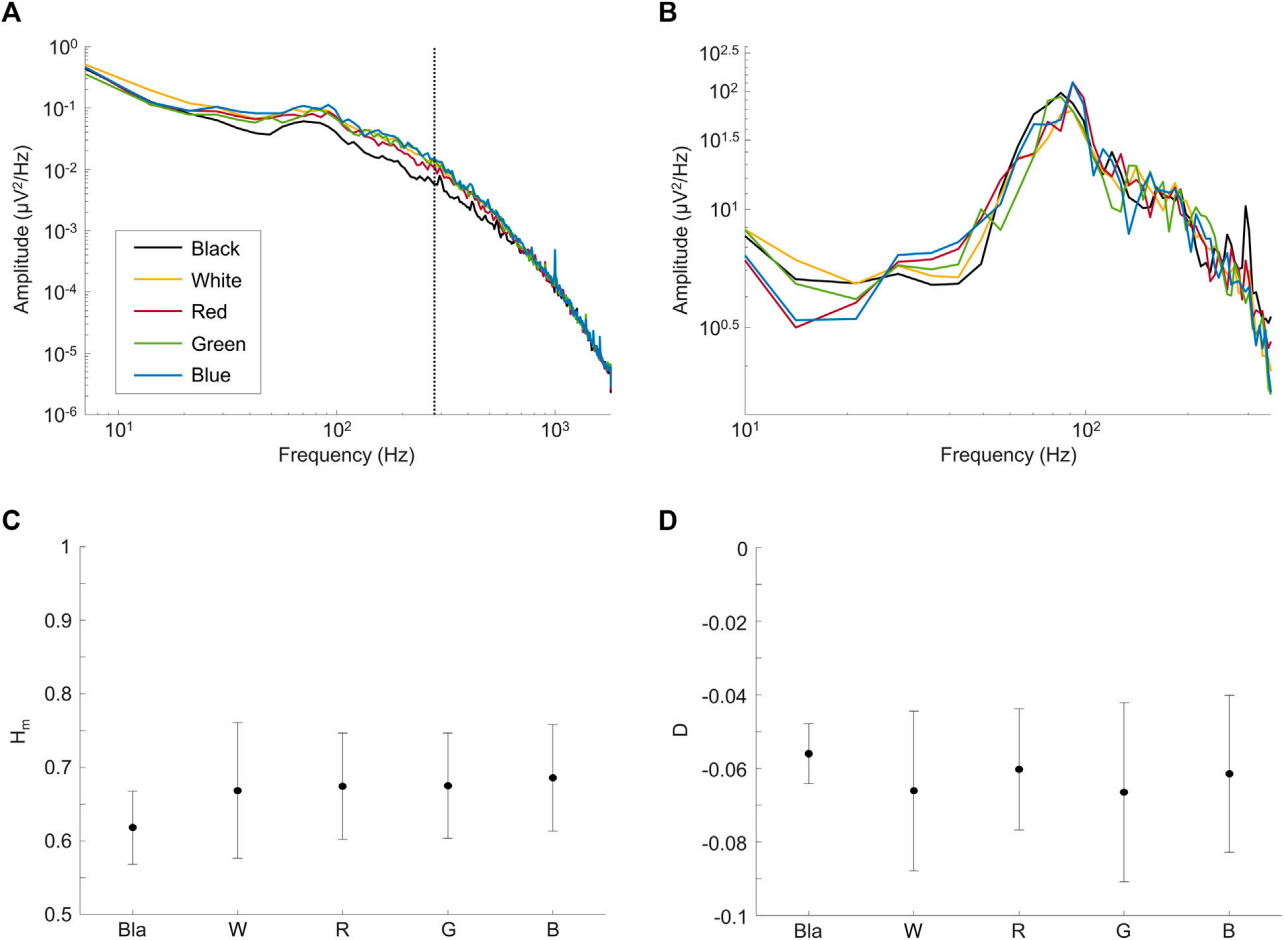
Comparing rsERGs at background light colors. Mean frequency spectrums of rsERGs obtained from different colored background lights at 30 cd m^−2^, i.e., black (no illumination; black tracings), white (yellow tracings), red (red tracings), green (green tracings) and blue (blue tracings). **(A)** Original mean frequency spectrums in log-log scale. Of note, the vertical dotted line shows the upper limit of data points used in the linear regression, i.e., data points from 7 Hz to 275 Hz. **(B)** Modified spectrums (zoomed) in log-log scale, with the $1/f^{\alpha }$ background activity removed. **(C,D)** Multifractal analysis on the colored rsERG signals. **(C)** Values ± STD of the main exponent $H_{m}$ . **(D)** Values ± STD of the dispersion index D .

In a first set of experiments, we aimed at determining whether the rsERG could be modulated by changes in the luminous environment. Results shown at Figures 3A,B reveal that rsERGs recorded following retinal exposure to luminous backgrounds of differing wavelengths all included an invariant peak in amplitude at ∼90 Hz, following a rise which started at ∼40 Hz. This invariance of the rsERG signal following exposure to different colored background conditions is also seen with the multifractal analysis in Figures 3C,D, where the H_m_ and D values are not statistically different from each other, for any of the tested conditions (*p* > 0.05). Of note, the dark (black) background seems to differ from the other conditions in Figures 3C,D, albeit not in a statistically significant way.

However, D being significantly different from 0 (and negative; Figure 3D; *p* < 0.05) in all test conditions confirms our use of the multifractal formalism. Furthermore, the H_m_ value stays ∼0.65 for all conditions (Figure 3C), which is in the range of values categorizing a long-range dependence in the signal (i.e., when H_m_>0.5).

**FIGURE 4:**
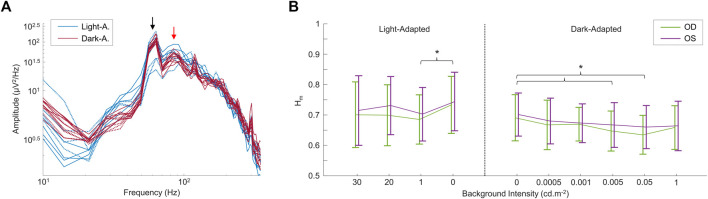
Comparing rsERGs at different background light intensities and retinal adaptation states. **(A)** Modified (removed $1/f^{\alpha }$ background) and zoomed mean frequency spectrum depicted on a log-log scale for all light- (blue tracings) and dark-adapted (red tracings) rsERGs obtained from different background luminance. For the detailed tracings for each luminance, Supplementary Figure S2. Of note, both eyes are shown separately, with OD shown in full lines and OS in dashed lines. The red arrow shows the 90 Hz peak, while the black arrow shows the 60 Hz contamination, which was greater in these recordings than in those from Figure 3 **(B)** Values ± STD of the main exponent $H_{m}$ , for both light- and dark-adapted rsERGs at different background light intensities, and for both eyes. Statistically significant differences (*p* < 0.05) between values in both eyes are shown with an asterix (*).

In a second set of experiments, we modulated the intensity of the background light both in light adaptation and following a period of dark-adaptation of 20 min, the results of which are shown in Figure 4. Again, the frequency spectrum shows a peak at ∼90 Hz (see red arrow in Figure 4A), that does not change either with light intensity or retinal adaptation conditions (see also Supplementary Figure S2). Of note, contrary to changes in wavelength, changes in intensity of the background light had an effect on the H_m_ values (but not in the D values; not imaged) between some of the conditions tested, as shown in Figure 4B. In the light-adapted condition, only the 0 cd m^−2^ and 1 cd m^−2^ background light conditions differed (*p* < 0.05) between each other. For the dark-adapted conditions, the complete dark condition (0 cd m^−2^) differed (*p* < 0.05) with the 0.005 cd m^−2^ and the 0.05 cd m^−2^ background light conditions in both eyes. Finally, the data was not significantly different (*p* > 0.05) between OD and OS, in both the frequency spectrums and the multifractal analysis values.

### 3.3 The resting-state ERG reveals intrinsic and induced retinal components in the Flash evoked ERG

**FIGURE 5:**
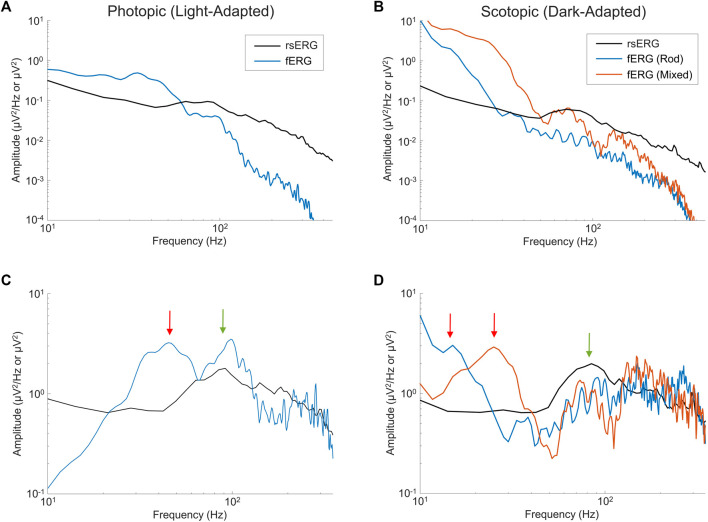
Comparing fERGs to rsERGs. Mean frequency spectrums depicted on a log-log scale for light- **(A,C)** and dark-adapted **(B,D)** rsERG and fERG signals. Of note, the fERG signal amplitudes were divided by 10 for better visual comparisons in **(A,B)**. Note a difference between the rsERG and fERG frequency spectrum units (µV/Hz vs. µV), as 2 different techniques were used (as explained in the methods). **(A,B)** Original spectrums. **(C,D)** Modified spectrums (zoomed), with the $1/f^{\alpha }$ background activity removed. Red arrows show peaks at low frequencies (about 15–45 Hz) present only in the fERGs, while green arrows show peaks at higher frequencies (about 80–120 Hz) present in both rsERGs and fERGs.

As defined above, the rsERG differs from the fERG as the latter requires a flash of light to be visualized while the former does not; our claim being that the rsERG represents the intrinsic electrical signal generated by the retina. We therefore aimed at determining if, in the fERG, we could also identify this intrinsic component and, conversely, what part of the fERG is artificially created as a result of the use of a flash. In Figure 5 are compared light- (Figures 5A,C) and dark-adapted (Figures 5B,D) fERG and rsERG raw frequency spectrums, while in Figures 5C,D the spectrums are shown without the $1/f^{\alpha }$ background activity in order to enhance the underlying periodic components.

In the light-adapted condition shown in Figure 5C, the ISCEV standard (McCulloch et al., 2015) photopic fERG (flash: 3 cd s m^−2^, background light: 30 cd m^−2^) was compared to the rsERG recorded while facing the 30 cd m^−2^ white light background. The green arrow points at the ∼95 Hz (rsERG: 90.6 ± 4.9 Hz; fERG: 98.9 ± 6.5 Hz) components which is common to both retinal responses, while the red arrow points at a ∼45 Hz (45.2 ± 5.4 Hz) seen only in the fERG response.

Figure 5D shows the ISCEV standard scotopic fERG (pure-rod response flash: 0.005 cd s m^−2^; mixed-response flash: 1 cd s m^−2^, background light: 0 cd m^−2^) compared to the 0 cd m^−2^ background light (total dark) rsERG. Similar to the light-adapted condition, the dark-adapted responses share a similar high frequency component (see green arrow) oscillating between ∼75 and 90 Hz (rsERG: 83.5 ± 10.3 Hz; fERG rod: 91.5 ± 9.9 Hz; fERG mixed: 75.0 ± 3.3 Hz), while the fERG signals also conceal another frequency component (see red arrows) at 15.9 ± 1.9 Hz (pure-rod) and 25.0 ± 1.7 Hz (mixed).

### 3.4 The effect of retinal disease on the resting-state ERG

**FIGURE 6:**
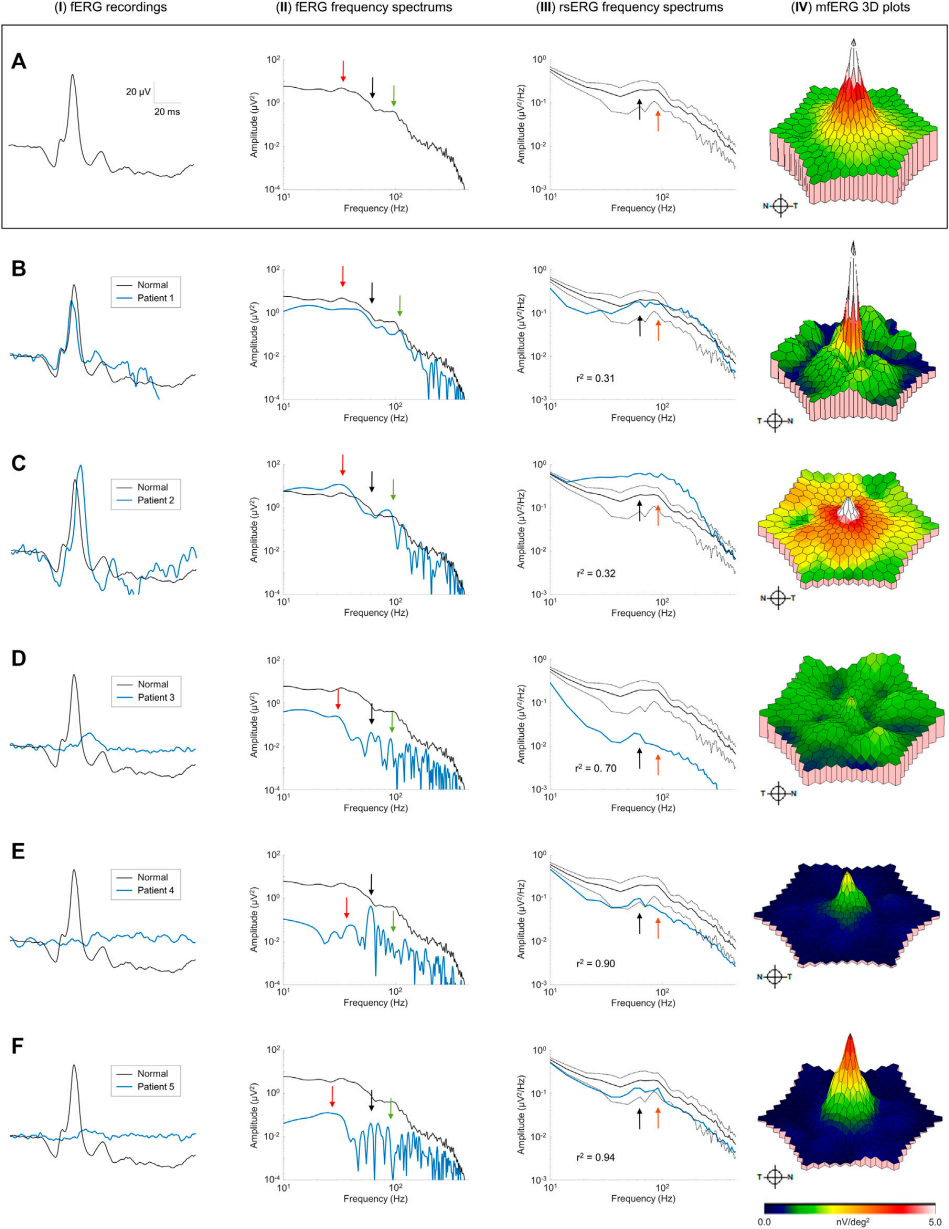
Pathological cases and the rsERG. Examples of pathological cases (blue traces) compared to normal subjects (black traces). Row **(A)** shows examples of normal data, while each subsequent row **(B–F)** shows a different patient. The first column (**I**) shows the ISCEV standard photopic fERG (flash: 3 cd s m^−2^, background light: 30 cd m^−2^) recordings, with the associated frequency spectrums shown in the second column (**II**). The light-adapted 30 cd m^−2^ white background light rsERG frequency spectrums are shown in the third column (**III**), with the normal subject mean ± standard deviations shown in black dashed lines. The last column (**IV**) shows the mfERG 3D plots, with the full mfERG data shown in Supplementary Figure S3. Black arrows show the 60 Hz component. Red arrows show the fERG slow component. Green arrows show the fERG fast component. Orange arrows show the rsERG 90 Hz fast component. The r^2^ value between the normal and pathological rsERG frequency spectrums are shown on the bottom left corner of each figure. All r^2^ values were significant (*p* < 0.05).

Finally, we recorded the rsERG in patients affected with known retinopathies, to assess whether it could be used to detect a disease state. Figures 6B–F shows five examples of data collected from patients affected with selected retinal pathologies (compared with normal subjects in Figure 6A). To have a more complete picture of the retinal function of these patients, fERGs (which measures the retinal function over its entire surface) and multifocal ERGs (mfERGs; giving a topographical assessment of macular function) were also recorded and analyzed. In order to better appreciate differences in the shapes of the rsERG frequency spectrums, these were normalized to the value at the first data point (i.e., 7 Hz), and a correlation was calculated between the rsERG frequency spectrums of normal and diseased retinas. Interestingly, as shown in Figure 6, while the amplitudes of the fERGs gradually progress (Figures 6B–F) from normal to extinguished, the r^2^ value of the rsERG varies in the opposite way [i.e., starting from a low value (at r^2^ = 0.31) to progressively reach a closer to normal value (at r^2^ = 0.94)].

Individual patient data also reveals interesting trends. In patients #1 and #2 (Figures 6B,C), the fERG responses are within the normal amplitude and peak time ranges. Similarly, the corresponding FFTs (Figures 6B,C-II) also yield a normal frequency distribution with key frequency components present in both responses (see red and green arrows). In contrast, the rsERG frequency spectrums (Figures 6B,C-III) differ markedly from normal, as reflected with the low r^2^ values (Patient #1: r^2^ = 0.31; Patient #2: r^2^ = 0.32). This change in morphology is most obvious with Patient #2 (Figures 6C-I), where the 40 Hz trough that normally precedes the 90 Hz peak is lacking, thus making the latter less prominent. Patient #2 also has a reduced macular function as seen with the low mfERG amplitude (Figures 6C-I), while Patient #1 also had an altered mfERG response, with noisy waveforms (Supplementary Figure S3B), resulting in patches of low amplitude on the 3D plot in Figures 6B-I.

The other three patients (#3–5) all had significantly reduced fERGs (Figures 6D–F-I), resulting in significantly reduced amplitudes (by 1 to 2 log-units) of their FFT frequency spectrums (Figures 6D–f-II). In contrast, while the rsERGs of Patients #4 and #5 were within the normal range in amplitude and morphologies (as evidenced with the high r^2^), that of Patient #3 (Figures 6D-I) was significantly different from normal, both in rsERG frequency spectrum amplitude (significantly below the normal limits), and its aperiodic component (i.e., $1/f^{\alpha }$ background activity). This patient also had a nearly extinguished mfERG (Figures 6D–I), while subjects #4 and #5 had a preserved foveal peak (Figures 6E,f-IV). However, all r^2^ values showed a high level of correlation (r^2^ > 0.7), meaning that the morphologies of these patients’ rsERG frequency spectrums were similar to that of the normal population, with a small trough at about 40 Hz and a peak at about 90 Hz (Figures 6D–f-III).

## 4 Discussion

The purpose of this study was to develop a method to record and analyze the resting-state ERG (or rsERG) which is defined as the intrinsic (or spontaneous) electrical activity generated by the retina. The rsERG thus contrasts with the better-known flash ERG (or fERG) in that the former does not rely on a synchronizing stimulus (such as a flash of light) for its production while the latter does. Thus, the rsERG parallels the rsEEG in that both signals are obtained without use of tasks or stimuli to generate a response but rather assess the intrinsic/endogenous neuronal activity (Snyder and Raichle, 2012; Babiloni et al., 2020; Li et al., 2020).

Given that the rsERG does not have a clearly distinguishable waveform like the fERG, we opted for spectral analysis approach similar to that used in EEG (Zorick and Mandelkern, 2013; Voytek et al., 2015a; Donoghue et al., 2020)*.* Donoghue et al. (2020) showed that both periodic and aperiodic components need to be extracted from the frequency spectrums of biopotentials. The periodic components, which are defined as peaks in specific frequency bands of the EEG, are usually associated to physiological, cognitive or behavioral activities (Voytek et al., 2015a; Engel et al., 2001). On the other hand, the aperiodic component represents the underlying background activity which recruits wider frequency bands and whose power decreases with increasing frequency, following a $1/f^{\alpha }$ power law dynamic. This activity is said to reflect a balance between excitatory and inhibitory neuronal activities (Gao et al., 2017; Molina et al., 2020), which can change with age (Voytek et al., 2015b), but also with cerebral activity, cognitive or motor (Miller et al., 2009; He, 2014). As with the brain, inhibitory and excitatory activities also exist in the retina, most notably the ON-OFF pathway (Light onset/offset responses) (Howarth, 1961; Gauvin et al., 2017). This aperiodic component is not solely described by a single scaling exponent α (as our multifractal D value was found to be significantly different from 0 in all conditions), but rather by a range of such exponents which reflect the changing power laws present in the frequency spectrum, hence our use of a multifractal analysis.

**FIGURE 7:**
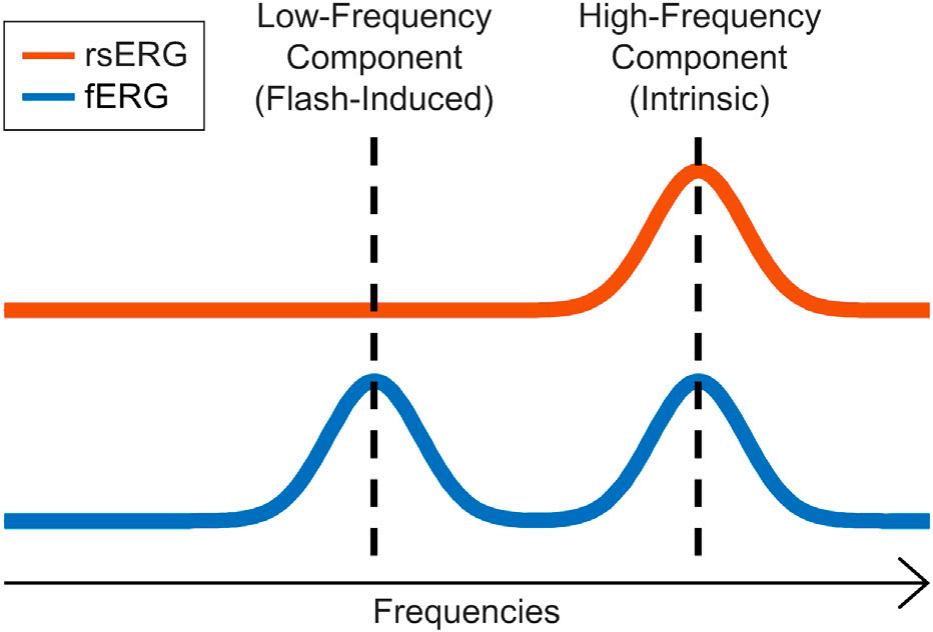
rsERG vs. fERG representation. Illustration of the intrinsic (high-frequency) and induced (low-frequency) components in the fERG and rsERG.

In our first set of analysis, we found that irrespective of the background luminance to which the retina was exposed, a 90 Hz frequency peak remained nearly invariable, as evidenced at Figures 3A,B and Figure 4A, where the different frequency spectrums are overlapping. This either suggests that this periodic component is the frequency signature of all rsERGs, irrespective of how they are generated or that the recording conditions that we tested were not distinct enough to generate significantly different frequency peaks, should this concept exist. It is however of interest to note that the results obtained from our selected patients would suggest that the periodic component of the rsERG can vary as a result of a given retinal disease process. The latter finding would support a diagnostic value to the rsERG. It is of interest to note that this high frequency component of the rsERG is also observed in the frequency spectrums of the photopic and scotopic fERG (Figure 5). Given its alleged intrinsic nature, this would suggest that the flash stimulus is not essential to the presence of this high-frequency activity in the fERG response. In contrast, our demonstration that the low-frequency component of the fERG (which is absent in the rsERG) varies with flash intensity (increasing the flash intensity increases the peak frequency), would suggest that the latter is a man-made (i.e., artificial) fERG component (i.e., flash-induced; Figure 7 for explanatory illustration). Furthermore, Dickey et al. (2022) have recently shown that a widespread 90 Hz ripple is present, during sleep and waking, in intracranial recordings in humans, which are theorized to synchronize activity between cortical regions and help bind information. The correspondence between this component and the one we report here at exactly the same frequency (i.e., 90 Hz) is probably more than coincidental and warrants additional research to be better understood.

Our results further revealed that the different luminous environment tested altered only the aperiodic component of the rsERG of some conditions compared to dark (no light; Figure 4B). Adding only a small quantity of light can modify the aperiodic component of the rsERG, while adding a larger amount will revert this effect. The latter would suggest a modulatory effect of light on the aperiodic component, a feature possibly associated with the ON-OFF paradigm; a concept in line with the behavior of the aperiodic component of the EEG which is said to be a reflection of the balance between excitation/inhibition (i.e., ON-OFF) activities (Gao et al., 2017; Molina et al., 2020).

Finally, our brief survey of pathological cases revealed that combining the recording of the rsERG with the fERG could yield significant information given that they are not always similarly affected, just like combining the mfERG reveals further information often not revealed by exclusively using the fERG. Although our selection of retinopathies could suggest that the retinal disorder only contributes to the electrophysiological phenotype that we measured, we cannot, at this point, exclude the possibility that other pathophysiological processes could also contribute to the electroretinographic (fERG, mfERG and/or rsERG) signature of an individual. We found that when the mfERG was almost extinguished, the aperiodic component of the rsERG was also abnormal [as in patients #2 (normal fERG) and #3 (residual fERG)], while normal or even residual macular function (i.e., only central rings of the mfERG remaining) was associated with normal rsERGs [as in patients #4 and #5 (both had extinguished fERGs)]. Of note, it is at the level of the fovea that we find the highest density of cone photoreceptors (Curcio et al., 1990). We claim that this peak in photoreceptor density, combined with the 1-1-1 ratio between cone-bipolar cells-ganglion cells observed at the fovea (Bringmann et al., 2018), generates an electric dipole moment of a magnitude significantly larger than that measured (for the same surface unit) anywhere else in the retina, thus explaining the higher contribution of the fovea to the genesis of the rsERG compared to the rest of the retina. The latter concept needs however to be further investigated.

In conclusion, the purpose of this study was to investigate possible to record the intrinsic electrical activity of the retina and, if so, if the rsERG could add a new dimension to the diagnosis and characterization of retinal disorders. Results obtained from our normal cohort clearly show that the rsERG can be recorded and its analysis does yield reproducible results. Furthermore, despite the small sample size, our selection of retinal pathologies, indicates that the rsERG (when compared to findings obtained with the flash and multifocal ERGs) does add a new diagnostic information that could be of some use in the differential diagnosis of retinopathies.

## 5 Statements of human rights

All procedures performed on human participants were done so in accordance with the ethical standards of the Institutional Review Board of the McGill University Health Center and in accordance with the ethical standards as laid down in the 1964 Declaration of Helsinki and its later amendments or comparable ethical standards.

## 6 Informed consent

All subjects freely consented to participate in this study, and an informed consent was obtained from all participants included in the study.

## Data Availability

The raw data supporting the conclusion of this article will be made available by the authors, without undue reservation.
