# Supplementary material for: Resting state electroretinography: An innovative approach to intrinsic retinal function monitoring
Source: Front Physiol. 2022 Aug 26;13:931147. doi: 10.3389/fphys.2022.931147 (PMC9462834; doi:10.3389/fphys.2022.931147)
Supplement: Supplementary file 1 [file DataSheet1.pdf]

Supplementary Material

1 ANNEXES

1.1 ANNEX I: Scale-Invariance and $1/f^{\alpha}$ Power Spectrum

We can characterize a scale-free signal by a self-similar process x(t), at a scale a, with:

$$x(at) \approx a^{H}x(t) \tag{A.1}$$

Following this equality and if the Hurst exponent $H > 0$, a signal $x(t)$ would reproduce a similar process at all scales a, whether it is zoomed in or out, with each scale having a normalized amplitude given by the value of the exponent H. The analysis of such self-similar processes can be challenging due to their non-stationarity, but we can describe a subclass containing stationary increments, defined as $x_{\theta}(t) = x(t+\theta) - x(t)$ for a given time delay θ and $x_{\theta}(t) \approx x_{\theta}(0)$. Then replacing $x(t)$ with $x_{\theta}(t)$ reproduces the self-similar property. The statistical moments (or structure function $S_q(j)$) of these stationary increments can then be given by:

$$S_q(j) \stackrel{\text{def}}{=} E[|x_{\theta}|^{q}] \sim \theta^{qH}, for\ q > -1 \tag{A.2}$$

For large time delays τ, the autocovariance of these increments behave as:

$$E[x_{\theta}(t-\tau)x_{\theta}(t)] \sim H\gamma\theta^{2}\tau^{\gamma-1}, \gamma = 2H - 1 \tag{A.3}$$

From here, the process will have one of 2 possible fluctuation types in the time series, according to the value of H (Flandrin, 1992). If H is between 0.5 and 1, then the process shows persistence or long-range dependency. If on the other hand H is between 0 and 0.5, then the process shows anti-persistence or more complexity. Furthermore, the PSD of the increment process will follow approximately a $1/f^{\gamma}$ behavior, while the power spectrum of its original signal $x(t)$ will follow approximately a $1/f^{\gamma+2}$, thus about $1/f^{\alpha}$ with $\alpha = 2H + 1$.

1.2 ANNEX II: Wavelet Leaders calculation and analysis of scale-Invariance

1.2.1 Wavelet analysis for scale-invariance

As shown in Annex I, we can define the SI with increments, which are fluctuations at a specific time scale θ. We can analyze this in terms of discrete wavelet coefficients, defined as:

$$d(j,k) = \int_{-\infty}^{+\infty} x(t)2^{\frac{-j}{2}}\psi(2^{-j}t - k)dt \tag{A.4}$$

where each time-frequency coefficient $d(j,k)$ is obtained by transforming the signal $x(t)$ by a wavelet ψ, which is itself scaled by 2^{-j} and translated by $2^{j}k$ (Daubechies, 1992b, Daubechies, 1992a). By doing so, the increments $x_{\theta}(t)$ are obtained at the scale $\theta = 2^{j}$, with the wavelet $\psi(t) =$

$\delta(t-1)-\delta(t)$. Thus, with the wavelet coefficients $d(j,k)$ of signal $x(t)$ and with the scale $a = 2^j$, we can rewrite eq. (1) and (2) as:

$$d(j,k) \approx 2^{jH} d(0,k) \quad \text{(A.5)}$$

$$S_q(j) \stackrel{\text{def}}{=} E[|d(j,\cdot)|^q] \sim 2^{jqH} \quad \text{(A.6)}$$

This generalized the process, giving it a unique and exact H exponent, but a range of many such exponents $h(t)$ could be needed to describe the process. Instead of a single value H, a function $\zeta(q)$ can be needed to describe the self-similar behavior in the signal. The multifractal formalism uses this function (Muzy et al., 1994), and rewrites the structure functions as $S_q(j) \sim 2^{j\zeta(q)}$. A monofractal process would have single H value, thus $\zeta(q) = qH$, and we retrieve eq. (5).

This work will consider a multifractal framework, with the quadratic expansion $\zeta(q) = qH_m + 1/2Dq^2$ to generalize the signal's SI. Thus, the goal is to find the most prevalent exponent H termed H_m and the dispersion D of these exponents around H_m. If $D = 0$, then the signal is monofractal: there is no dispersion, i.e., a single H exponent exists. If on the other hand $D < 0$ [D cannot be positive since $\zeta(q)$ needs to be convex (Wendt and Abry, 2007)], then the signal is multifractal and thus increases in complexity.

1.2.2 Wavelet Leaders calculation

The WLBMF Matlab toolbox (Wendt et al., 2007) estimates the set of Hölder exponents by estimating a local power law from a modified discrete wavelet transform (DWT) termed the Wavelet Leaders (Jaffard, 2004, Jaffard et al., 2006, Wendt and Abry, 2007, Wendt et al., 2007). First, the DWT was calculated on the signal at hand as in eq. (3). Second, the DWT was modified to obtain the Wavelet Leader coefficients $L(j,k)$. Each coefficient was compared to its neighbors in a specific set:

$$L(j,k) = sup_{\lambda' \subset 3\lambda} |d, \lambda'| \quad \text{(A.7)}$$

where the set λ is called a dyadic interval, defined as:

$$\lambda = \lambda j,k = [k2^j, (k+1)2^j) \quad \text{(A.8)}$$

Thus, each $d(j,k)$ coefficient becomes the maximum value of a set of neighboring coefficients belonging to its dyadic interval and the 2 others adjacent to it, such that:

$$3\lambda_{j,k} = \lambda_{j,k-1} \cup \lambda_{j,k} \cup \lambda_{j,k+1} \quad \text{(A.9)}$$

This modification of the DWT regularizes the time-frequency scalogram, with coefficients increasing with the analyzed scale (frequency), thus making it possible to calculate the Hölder exponent h at each time point t_0 as a local power law behavior over a set of scales j, such that:

$$L(j, t_0) \sim C2^{jh(t_0)} \quad \text{(A.10)}$$

1.2.3 Log-Cumulants

To describe the signal using the WLBMF toolbox, as proposed by Wendt et al. (2007), we used what is called log-cumulants (specifically the first 2: c_1 and c_2). In this study, these were

referred as the main Hölder exponent H_m (i.e., c_1), and the dispersion index D (i.e., c_2), that accounts for the multifractality of the process, i.e., the dispersion in Holder exponents. The necessary concavity of the scaling exponents with respect to the statistical orders was used to validate the multifractal model (Wendt and Abry, 2007). Examples of the multifractal spectrums obtained can be found in **suppl. Fig. S4**. The scales j between 2 and 9 (corresponding to a range of frequencies going from 3.5 Hz to 901.5 Hz) were used, as well as moments of order q going between -5 and 5, using 49 bootstrap resamples of wavelet leaders. The mother wavelet chosen was Daubechies 4 (with 4 vanishing moments).

2 Supplementary Figures and Tables

2.1 Supplementary Figures

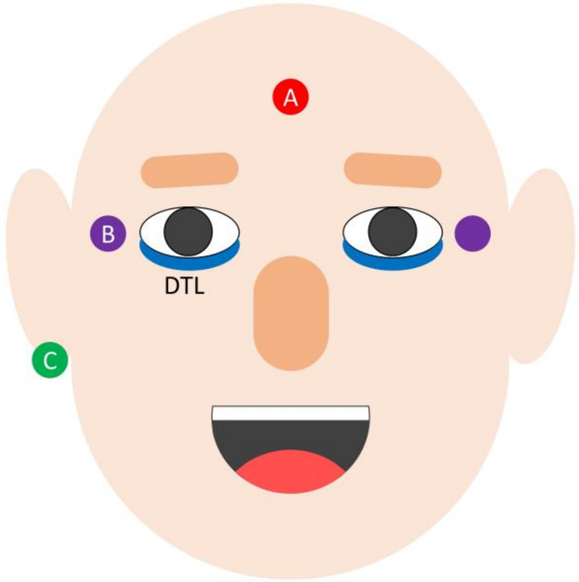

Supplementary Figure 1. Representation of an individual's electrode setup. Electrodes were placed in four different locations on a subject's head for this study. For the fERG and rsERG, the active electrodes (**DTL** in blue) were placed deep in the inferior conjunctival bags of each eye, the reference electrodes (**B** in purple) were placed on the externa canthi and the ground (**A** in red) was placed on the forehead. For the EEG setup, the active electrode was on the forehead (**A** in red), the reference was on the right external canthus (**B** in purple) and the ground was on the right earlobe (**C** in green).

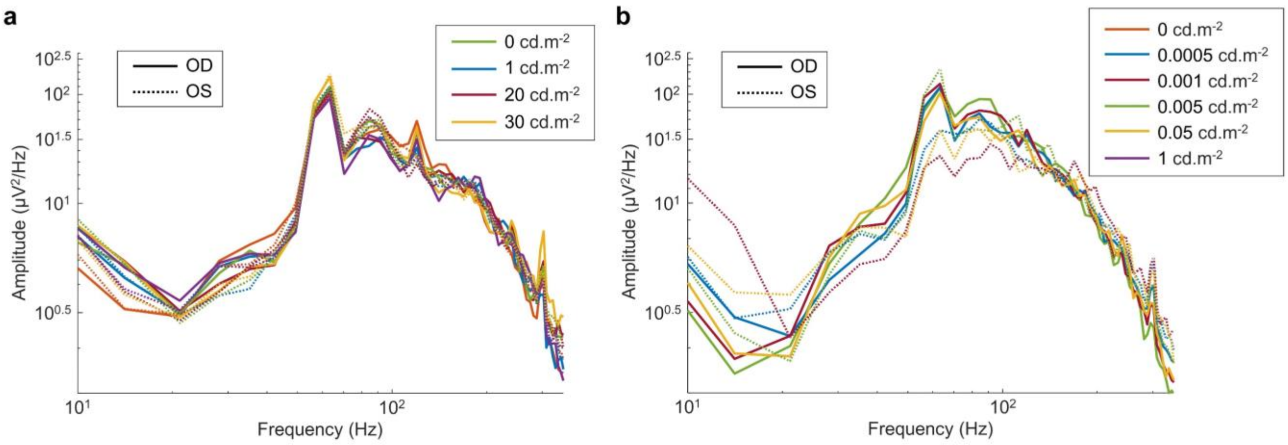

Supplementary Figure 2. Detailed light- and dark-adapted rsERG frequency spectrums. Modified (removed $1/f^{\alpha}$ background) and zoomed mean frequency spectrums depicted on a log-log scale for light- (**a**) and dark-adapted (**b**) rsERGs obtained from different background illuminations, in both eyes (OD and OS).

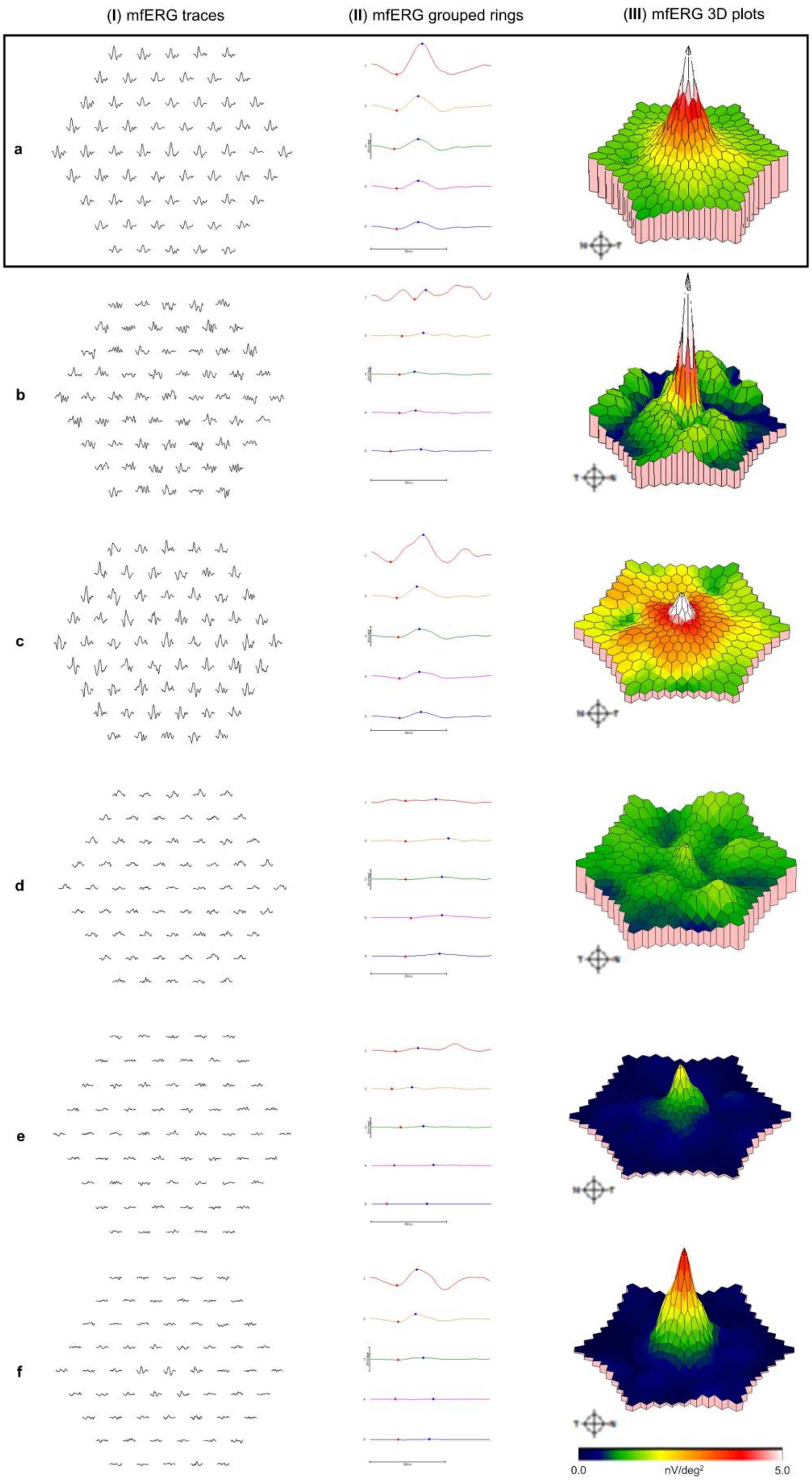
(I) mfERG traces
(II) mfERG grouped rings
(III) mfERG 3D plots
a
b
c
d
e
f
0.0
nV/deg^2
5.0

Supplementary Figure 3. Detailed mfERGs of all patients. mfERGs of the pathological cases shown in **Fig. 4** and **Table 1**. Row **a** shows an example of a normal subject, while each subsequent row (**b-f**) shows a different patient, as in **Fig. 4**. Columns show data as follows: (**I**) mfERG traces; (**II**) mfERG group tracings for each ring; (**III**) mfERG 3D plots.

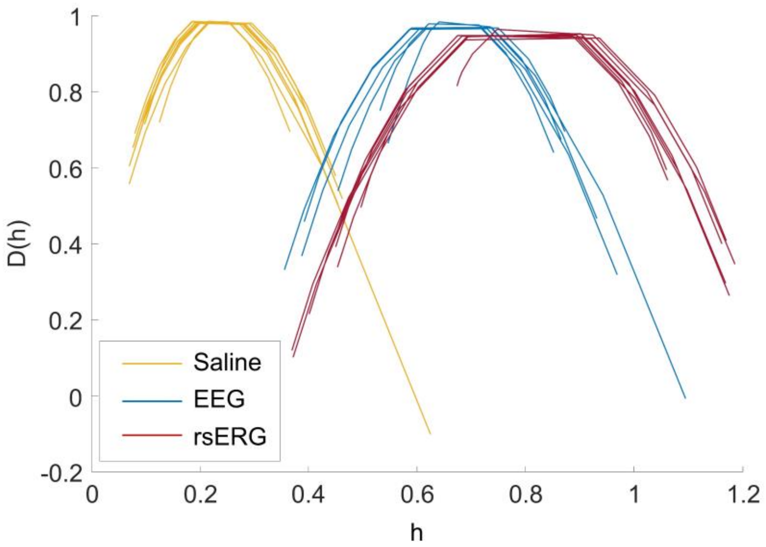

Supplementary Figure 4. Multifractal spectrums of all three types of signals used in the study. Multifractal spectrums D(h) are shown for individual epochs recorded (i.e., multiple spectrums shown per signal type). EEG and rsERG examples are of all epochs for one subject, given as an example.
